# Impact of multidisciplinary cardiology rounds on a cardiac ICU: a prospective cohort study

**DOI:** 10.1186/cc14667

**Published:** 2015-09-28

**Authors:** Rodrigo MV Melo, Amilton S Junior, Fabio Gomes, Fernando Colombari, Fernando Zampieri

**Affiliations:** 1 Intensive Care Unit, Hospital Alemao Oswaldo Cruz, Bela Vista, São Paulo, São Paulo, Brazil

## Introduction

Quality improvement is an important activity for all members of an interdisciplinary cardiology critical care team.

## Objective

To evaluate whether multidisciplinary quality improvement in cardiac critical care, focused on a daily routine of rounds, protocol standardizations, and under the leadership of the same attending cardiologist in charge of coordinating a team, could produce better outcomes and resource utilization.

## Methods

Prospectively collected data for consecutive patients who were admitted to a nine-bed cardiac intensive care unit (CICU) in two periods: January-June 2013 and January-June 2014. In the first period (control group) the patients were evaluated by common CICU routine, each day attended by a different intensivist physician, with no standardization of the multidisciplinary approach. Between the two periods there was a 6-month multidisciplinary training. In the second period (intervention group) the same cardiologist and multidisciplinary team made the daily routine rounds, with standardizations of managements and evidence-based care. Demographics and outcomes (mortality, time of ICU stay and mechanical ventilation time) were compared.

## Results

A total of 610 patients were evaluated in the period of the study, 314 (51.4%) in the control group and 296 (48.6%) in the intervention group. Both groups were well matched for demographics: intervention and control group respectively, mean age of 68.9 (±14.7) vs. 70.9 (±13.2), *p *= 0.08; admission after cardiac surgery 21 (7.1%) vs. 26 (8.3%), *p *= 0.40; admission after percutaneous interventions 60 (20.3%) vs. 59 (18.8%), *p *= 0.55. The mean predicted mortality assessed by the simplified acute physiology score 3 (SAPS-3) and the Charlson comorbidity index were similar in both groups: intervention and control respectively, 43.1 (±13.1) vs. 42.8 (±12.9), *p *= 0.66 and 1.91 (±2.1) vs. 1.90 (±2.2), *p *= 0.97. Despite this, the mean ICU stay was lower in the intervention group as compared with the control group, 2.5 (±3.4) vs. 3.4 (±3.8) days, *p *= 0.003; as was the mean mechanical ventilation time 0.84 (±0.16) vs. 4.16 (±1.47), *p *= 0.005. The 30-day mortality was 11 (3.7%) vs. 15 (4.7%), RR 0.79, 95% CI 0.64-3.03, *p *= 0.40. After multivariate analysis, there were no changes in the results.

## Conclusion

A focused cardiac critical care management on a CICU, based on a multidisciplinary approach and daily rounds performed by the same cardiologist, reduced the CICU stay and mechanical ventilation time, with the same mortality rates. This action could help improve resource utilization.

